# Prevalence and risk factors for anemia severity and type in Malawian men and women: urban and rural differences

**DOI:** 10.1186/s12963-017-0128-2

**Published:** 2017-03-29

**Authors:** Aishatu L. Adamu, Amelia Crampin, Ndoliwe Kayuni, Alemayehu Amberbir, Olivier Koole, Amos Phiri, Moffat Nyirenda, Paul Fine

**Affiliations:** 1 Bayero University Kano, Community Medicine, Kano, Nigeria; 2 London School of Hygiene and Tropical Medicine, London, United Kingdom; 3 Malawi Epidemiology and Intervention Research Unit, Karonga, Malawi

**Keywords:** Anemia severity, Anemia/epidemiology, Men, Women, Adult, Risk factors, Urban, Rural

## Abstract

**Background:**

The global burden of anemia is large especially in sub-Saharan Africa, where HIV is common and lifestyles are changing rapidly with urbanization. The effects of these changes are unknown. Studies of anemia usually focus on pregnant women or children, among whom the burden is greatest. We describe prevalence and risk factors for anemia among rural and urban men and women of all ages in Malawi.

**Methods:**

We analyzed data from a population-wide cross-sectional survey of adults conducted in two sites, Karonga (rural) and Lilongwe (urban), commencing in May 2013. We used multinomial logistic regression models, stratified by sex to identify risk factors for mild and moderate-to-severe anemia.

**Results:**

Anemia prevalence was assessed among 8,926 men (age range 18–100 years) and 14,978 women (age range: 18–103 years). Weighted prevalence levels for all, mild, and moderate-to-severe anemia were 8.2, 6.7 and 1.2% in rural men; 19.4, 12.0 and 7.4% in rural women; 5.9, 5.1 and 0.8% in urban men; and 23.4, 13.6 and 10.1% in urban women. Among women, the odds of anemia were higher among urban residents and those with higher socioeconomic status. Increasing age was associated with higher anemia prevalence in men. Among both men and women, HIV infection was a consistent risk factor for severity of anemia, though its relative effect was stronger on moderate-to-severe anemia.

**Conclusions:**

The drivers of anemia in this population are complex, include both socioeconomic and biological factors and are affecting men and women differently. The associations with urban lifestyle and HIV indicate opportunities for targeted intervention.

## Background

Anemia is a global public health problem, at any stage of life [1, 2]. A recent systematic analysis using national and subnational anemia survey data estimated a worldwide prevalence of 32.9% in all ages combined, contributing more years lived with disability than either depression or chronic respiratory diseases [3]. The bulk of the global anemia burden was driven by under-5 children and women [3].

Sub-Saharan Africa (SSA) has the highest regional prevalence of anemia with a slower decline over time than in other regions [1, 3]. Using WHO definitions, anemia is a severe public health problem among non-pregnant and pregnant women in SSA. Variations occur within the region, however, with highest prevalence and least improvement for subgroups in West and Central Africa [2]. A combination of population growth, aging population, infectious diseases and iron deficiency are driving the anemia burden in SSA [3]. The majority of published studies on anemia in SSA focus on children and pregnant women [4–9]. Men and elderly populations have received relatively little attention [10, 11].

Anemia has diverse consequences and different subgroups of the population have varying vulnerabilities to its complications, such as fatigue and congestive cardiac failure; the rate and severity increasing with the severity of anemia [12–14]. Conditions resulting in anemia act largely through either reduced red cell production or increased red cell loss, but the former is more often implicated in the etiology of severe anemia [15–17]. Although different risk factors influence anemia independently, they commonly exist concomitantly, making it challenging to single out a definitive cause especially in resource-poor settings where access to advanced diagnostic tools is limited [18, 19]. Important causes of anemia in developing countries include micronutrient deficiencies, infectious diseases, hemoglobinopathies, and maternal blood loss. In terms of presentation, iron deficiency anemia is typically microcytic hypochromic, and anemia from chronic diseases is normocytic normochromic, whereas macrocytic anemia is commonly associated with B12 and folate deficiencies, or drug and alcohol toxicities, though overlap is common [20–22]. Intricate relationships between economic, political, demographic, sociocultural and biological factors influence the patterns of underlying causes, vulnerability to, and distribution of anemia severity and consequences [8, 16, 19].

Distinction between different severity levels of anemia has been recommended for the appropriate monitoring of anemia in populations, especially as countries have intensified control efforts, shifting the burden to the lower end of the hemoglobin distribution [3, 16, 23]. For instance, a recent analysis of global anemia burden from 1990 to 2010 showed that although overall prevalence has declined for all severities, among men, prevalence of severe anemia appears to be increasing [3].

Prevalence of anemia in Malawi has been estimated from Demographic and Health Surveys (DHS) for under-5 children, pregnant women, and women of reproductive age. Men and women outside reproductive age are not included and study sample sites are scattered. According to the 2010 Malawi DHS, prevalence levels of mild, moderate, and severe anemia were 21.7, 5.8 and 0.6% for non-pregnant and 19.5, 17.8 and 0.2% for pregnant women [24].

In order to describe the prevalence and distribution of different severities and types of anemia, and to examine the risk factors for different severities of anemia, we analysed data from a large cross-sectional population study of cardio-metabolic risk factors in rural and urban adults in Malawi.

## Methods

A large population-wide cross-sectional survey was conducted in two sites by the Malawi Epidemiology and Intervention Research Unit (MEIRU) from 2013 to 2015. One site was rural – Chilumba, in southern Karonga district, northern Malawi. The rural study was nested within a demographic surveillance site (DSS) established by MEIRU’s forerunner, the Karonga Prevention Study, in 2002 [25–27]. The other site was an enumerated urban population, in Area 25 within Lilongwe city in central Malawi [25, 27].

### Study population

All permanently resident adults aged 18 years and above that were under surveillance or enumerated in either of the study sites were invited to participate, and informed written consent was sought. Reasons for non-participation include non-consent and being missed after three attempted visits to their respective households. Additionally, among those that consented some later either declined to provide blood samples or to have HIV tests done, or were not available at the time scheduled for venepuncture.

### Data collection

Data collection procedures have been previously described [27]. Sociodemographic information were obtained electronic questionnaires and checks were put in place to identify duplicates. Samples were transferred daily to the respective laboratories. Laboratory external quality assurance procedures were carried out by Thistles EQA. For internal control, samples were exchanged between study sites and tests repeated to ensure consistency [27]. HIV status was assessed using Determine and Bioline rapid test kits using serial protocols (conducted at the time of the survey, or in the rural DSS in recent linked studies), or was self-reported for those who were aware of their positive status. Anti-retroviral therapy (ART) status and duration were determined from self-report, and in the rural DSS from linked data on consenting clinic attenders.

### Anemia

Anemia status and severity were defined based on the WHO criteria for different hemoglobin cut-offs for men, non-pregnant, and pregnant women [28].

Based on recommendations by both the WHO and International Nutritional Anemia Consultative Group (INACG), we adjusted hemoglobin values for altitude and smoking [28–30]. This was done by subtracting 0.2 g/dl from individual hemoglobin values for participants in the Lilongwe study site (altitude: 1056 m above sea level). No adjustments are recommended for altitudes less than 1000 m, thus none were made for Karonga site subjects (altitude: 475 m). Smoking was adjusted for by subtracting 0.3 g/dl from individual hemoglobin values for current smokers (irrespective of how heavily an individual smoked). Twelve observations were excluded due to extreme and unlikely hemoglobin values (less than 4 g/dl for all, >18 g/dl for women, and >20 g/dl for men) [29]. We grouped anemia severity: no anemia, mild, and moderate-to-severe anemia for analysis. Any anemia was defined as hemoglobin values <13.0 g/dl in men, <12.0 g/dl in non-pregnant women, and <11.0 g/dl in pregnant women. Mild anemia was defined as hemoglobin values between 11.0 and 12.9 g/dl in men; 11.0 and 11.9 in non-pregnant women; and 10.0-10.9 in pregnant women. Moderate-to-severe anemia was defined as hemoglobin values <11.0 g/dl in men and non-pregnant women; and <10.0 g/dl in pregnant women.

### Exposure variables

Sociodemographic information and parity as well as pregnancy status were self-reported. Employment was categorized as not employed, subsistence farming/fishing, self-employed, or salaried. Individuals with body mass index (BMI) <18.5 kg/m2 were categorized as underweight. Wealth was categorized using a household asset value score (with urban and rural components appropriate for our setting) and divided into centiles across all urban and rural participants. Women with missing pregnancy status were treated as non-pregnant. Age, parity, and red cell indices [13] were categorized as follows:

- Age: 18–24, 25–34, 35–44, 45–54, 55–64, and ≥65 years
- Parity: 0, 1-2, 3-5, and ≥6 births.
- Mean cell volume (MCV): microcytosis (<80 fL), normocytosis (80–100 fL), and macrocytosis (>100 fL)
- Mean cell hemoglobin concentrations (MCHC): hypochromia (<32 g/dl), normochromia (32–36 g/dl), and hyperchromia (>36 g/dl)

### Statistical analysis

All analyses were done using STATA version 14.0 (Stata Corp, College Station, TX, USA). Weighted prevalence of anemia (any, mild, and moderate-to-severe) were calculated using the age structure of the respective underlying populations (rural and urban). Overall anemia prevalence (crude and weighted) and mean hemoglobin were first determined, followed by cross-tabulation of explanatory variables by anemia levels to explore unadjusted associations, and multinomial logistic (MNL) regression was used to obtain crude odds ratios (ORs).

MNL regression models were used for multivariable analyses of risk factors for varying severity of anemia, and adjusted ORs with their respective 95% confidence intervals (CIs) were calculated. The multivariable model was built up using a forward step-wise approach, but adjusting a priori for age and smoking in all models. Explanatory variables were retained if they confounded the ORs for any already-included variables (assessed by whether ORs changed by >10%) or there was at least weak association with anemia (*p*-value <0.2 from likelihood ratio test). If adding an explanatory variable to the model resulted in a change to the standard errors of the log odds ratios of already included variables by >20%, then the additional explanatory variable was assumed to be collinear and was excluded from the model.

We estimated the population attributable fraction (PAF) of HIV infection from the final multivariable model using the formula:

$$ \mathrm{P}\mathrm{A}\mathrm{F}=\mathrm{p}'\left(\uptheta \hbox{-} 1\right)/\uptheta $$

Where p’ was the proportion of persons with anemia who were HIV-positive or pregnant (in women) and θ was the adjusted OR from the multivariable model.

## Results

Of the 28,879 participants, 48% were from Karonga and 61.7% were women. Hematological data were available for 23,904 (84%). Descriptive characteristics of the study population are shown in Table 1. Mean age (±SD) was 36.0 ± 15.5 years in men, and 35.3 ± 14.6 years in women. Crude HIV prevalence was higher among female participants (9.3%) than males (6.2%).

**Table 1:** Background characteristics of study population

Variables	Total	Men	Women
	*N* = 28,879	*N* = 11,052	*N* = 17,827
Residence			
Rural (Karonga)	13,867	5,841(52.9)	8,026(45.0)
Urban (Lilongwe)	15,012	5,211(47.1)	9,801(55.0)
Age group (in years)			
18–24	8,169	3,299(29.9)	4,870(27.3)
25–34	8,947	3,023(27.4)	5,915(33.2)
35–44	5,385	2,133(19.3)	3,252(18.2)
45–54	2,900	1,153(10.4)	1,747(9.8)
55–64	1,722	681(6.2)	1,041(5.8)
> 65	1,756	754(6.8)	1,002(5.6)
Smoking history			
Never smoker	26,868	9,127(82.6)	17,741(99.5)
Past smoker	712	664(6.0)	48(0.3)
Current smoker	1,299	1,261(11.4)	38(0.2)
Alcohol use			
None	23,589	6,662(60.3)	16,927(94.9)
Light/irregular	4,052	3,236(29.3)	816(4.6)
Moderate/heavy	1,238	1,154(10.4)	84(0.5)
Education level			
No formal/illiterate	2,666	509(4.6)	2,156(12.1)
Standard	11,238	3,739(33.8)	7,499(42.1)
Secondary	12,350	5,503(49.8)	6,847(38.4)
Tertiary	2,625	1,301(11.8)	1,324(7.4)
Occupation			
Unemployed	10,941	3,101(28.1)	7,840(44.0)
Farming/Fishing	8,710	3,390(30.7)	5,320(29.8)
Self-employed	4,696	1,910(17.3)	2,786(15.6)
Salaried worker	4,532	2,651(24.0)	1,881(10.6)
Wealth quintile^a^			
1^st^ (lowest)	5,277	1,954(17.7)	3,323(18.6)
2^nd^	6,219	2,497(22.6)	3,722(20.9)
3^rd^	5,531	2,090(18.9)	3,441(19.3)
4^th^	6,658	2,502(22.6)	4,156(23.3)
5^th^ (highest)	4,957	1,911(17.3)	3,046(17.1)
Underweight^b^			
No	25,973	10,104(91.6)	15,869(94.6)
Yes	1,840	931(8.4)	909(5.4)
HIV status			
Negative	18, 673	7,067(63.9)	11,606(65.0)
HIV+	792	247(2.2)	545(3.1)
HIV+ on ART	1,562	443(4.0)	1,119(6.3)
Unknown	7,852	3,295(29.8)	4,557(25.6)
Currently pregnant			
No	16,817		16,817(94.3)
Yes	1,010		1,010(5.7)
Previous pregnancies^c^			
None	2,335		2,335(13.1)
1–2	5,310		5,310(29.8)
3–5	6,175		6,175(34.7)
≥ 6	3,981		3,981(22.4)

^a^ 237 missing ^b^ 1,066 pregnant women excluded from BMI ^c^ 26 missing

**Table 2:** Distribution of hemoglobin mean (SD) in men, non-pregnant women, and pregnant women stratified by age and HIV status

	Mean hemoglobin (SD)		
	Men	Non-pregnant women	Pregnant women
	n, mean (SD)	n, mean (SD)	n, mean (SD)
Total	8,926, 15.0(1.5)	14,211, 12.9(1.4)	767, 11.5(1.4)
Age group (years)			
18–24	2,593, 15.3(1.3)	3,635, 13.0(1.4)	317, 11.6(1.4)
25–34	2,398, 15.3(1.3)	4,614, 13.0(1.4)	369, 11.5(1.4)
35–44	1,736, 15.0(1.4)	2,763, 12.7(1.5)	81, 11.4(1.3)
45–54	967, 14.6(1.4)	1,531, 12.8(1.5)	0
55–64	592, 14.3(1.4)	912, 13.0(1.2)	0
> 65	640, 13.9(1.6)	756, 12.9(1.2)	0
HIV status			
Negative	6,588, 15.1(1.4)	10,429, 13.0(1.3)	561, 11.6(1.3)
HIV+ not on ART	218, 14.0(1.5)	476, 12.0(1.6)	19, 11.4(1.3)
HIV+ on ART	375, 14.2(1.7)	973, 12.4(1.6)	27, 10.6(1.7)
Unknown	1,745, 15.0(1.4)	2,333, 12.7(1.5)	160, 11.5(1.5)

### Anemia prevalence and distribution

A total of 3,874 participants were anemic and the overall sex, smoking, altitude, and pregnancy-adjusted prevalence of anemia in the study population was 16.2%. The respective crude prevalences of any, mild, and moderate-to-severe anemia were 7.4, 6.2 and 1.2% among men; 20.7, 12.2 and 8.5% among non-pregnant women; and 34.4, 22.2 and 11.9% among pregnant women. The mean (SD) hemoglobin (g/dl) was 15.0 (1.5) in men, 12.9 (1.4) in non-pregnant women, and 11.5 (1.4) in pregnant women. The mean (SD) hemoglobin concentration stratified by age group and HIV/ART status are shown in Table 2. The respective weighted prevalences of any, mild, and moderate-to-severe anemia were 8.2, 6.7 and 1.2% among rural men; 5.9, 5.1 and 0.8% among urban men; 19.4, 12.0 and 7.4% among rural women; and 23.5, 13.6 and 10.1% among urban women.

The distribution of morphological subtypes of anemia across severity by residence, sex, and pregnancy status are shown in Table 3. Across all subgroups, the proportion of microcytosis increased with severity of anemia. Microcytic anemia was higher with all levels of anemia among urban residents, while macrocytic anemia was more prevalent among men and rural residents.

**Table 3:** Distribution of anemia types by residence, sex and pregnancy status^a^

Anemia type	Mild anemia n(%)	Moderate-to-severe anemia n(%)
Rural (Karonga)	*N* = 1,196	*N* = 606
Microcytic	230(19.2)	259(42.7)
Normocytic	891(74.5)	317(52.3)
Macrocytic	75(6.3)	30(5.0)
Urban (Lilongwe)	*N* = 1,270	*N* = 802
Microcytic	508(40.0)	573(71.4)
Normocytic	735(57.9)	205(25.6)
Macrocytic	27(2.1)	24(3.0)
Men	*N* = 554	*N* = 110
Microcytic	134(24.2)	47(42.7)
Normocytic	375(67.7)	56(50.9)
Macrocytic	45(8.1)	7(6.4)
Non-pregnant women	*N* = 1,739	*N* = 1,207
Microcytic	555(31.9)	748(62.0)
Normocytic	1,135(65.3)	419(34.7)
Macrocytic	49(2.8)	40(3.3)
Pregnant women	*N* = 173	*N* = 91
Microcytic	49(28.3)	37(40.7)
Normocytic	116(67.0)	47(51.6)
Macrocytic	8(4.6)	7(7.7)

^a^ Only subjects with anemia

**Fig. 1:**
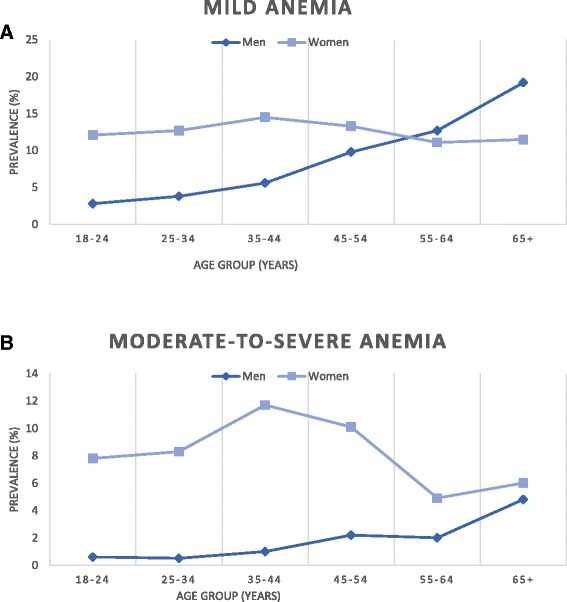
**a**. Prevalence of mild anemia by age in men and women. **b**. Prevalence of moderate-to-severe anemia by age in men and women

Among men, prevalence of both mild and moderate/severe anemia was lowest in the 18–24-year age group (Fig. 1a and b) and increased with age, particularly after 55 years of age. Among women, anemia prevalence peaked in the 35–44-year age group.

### Risk factors for anemia severity

Women in the urban area and those with higher education attainment were more likely to have anemia (Tables 4 and 5). Across the different occupation groups, farmers/fishermen had the highest anemia crude prevalence levels (any severity) among men, while among women, they had the lowest.

**Table 4:** Distribution of anemia severity and multinomial logistic regression showing crude ORs of risk factors for mild and moderate-to-severe anemia and respective p values in men

		Mild anemia		Moderate-to-severe anemia		
Variable	No of men	Prevalence (%)	Crude OR (95% CI)	Prevalence (%)	Crude OR (95% CI)	*P* value^1^
Residence						
Rural (Karonga)	4,997	6.8	1	1.5	1	
Urban (Lilongwe)	3,929	5.4	0.77(0.65–0.92)	0.9	0.58(0.39–0.87)	0.001
Age group (years)						
18–24	2,593	2.8	1	0.6	1	
25–34	2,398	3.8	1.36(1.00–1.86)	0.5	0.95(0.45–1.99)	
35–44	1,736	5.6	2.05(1.51–2.80)	1.0	1.85(0.93–3.69)	
45–54	967	9.8	3.83(2.80–5.25)	2.2	4.12(2.11–8.03)	
55–64	592	12.7	5.10(3.64–7.13)	2.0	3.97(1.85–8.53)	
>65	640	19.2	8.68(6.40–11.79)	4.8	10.65(5.71–19.88)	<0.001
Smoking status						
Never smoker	7,409	5.8	1	1.2	1	
Past	507	8.1	1.45(1.04–2.02)	1.8	1.60(0.80–3.20)	
Current	1,010	8.5	1.53(1.20–1.95)	1.6	1.43(0.83–2.45)	0.004
Alcohol use						
No	5,414	6.4	1	1.5	1	
Light/irregular	2,628	6.4	0.99(0.82–1.19)	0.9	0.57(0.36–0.90)	
Moderate/heavy	884	4.5	0.68(0.49–0.96)	0.5	0.29(0.10–0.78)	0.001
Education level						
No formal/illiterate	391	11.5	1.56(1.11–2.18)	3.6	2.69(1.46–4.96)	
Standard	3,092	7.9	1	1.4	1	
Secondary	4,406	5.0	0.62(0.51–0.74)	0.9	0.62(0.40–0.95)	
Tertiary	1,037	4.2	0.50(0.36–0.70)	1.2	0.78(0.41–1.48)	<0.001
Occupation						
Unemployed	2,505	5.6	1	1.3	1	
Farming/Fishing	2,886	7.6	1.38(1.11–1.72)	1.7	1.34(0.85–2.10)	
Self-employed	1,516	5.5	0.97(0.74–1.29)	1.4	1.08(0.62–1.89)	
Salaried worker	2,019	6.4	0.97(0.75–1.25)	0.5	0.35(0.16–0.73)	<0.001
Wealth quintile						
1^st^ (lowest)	1,544	7.6	1	1.4	1	
2nd	2,044	5.5	0.72(0.55–0.93)	1.6	1.13(0.65–1.96)	
3rd	1,695	7.5	0.99(0.76–1.28)	1.4	1.04(0.58–1.88)	
4th	2,032	6.0	0.78(0.60–1.01)	1.0	0.71(0.38–1.31)	
5^th^ (highest)	1,564	4.6	0.59(0.43–0.79)	0.8	0.59(0.29–1.18)	0.002
Underweight						
No	8,174	5.7	1	1.2	1	
Yes	740	11.5	2.17(1.70–2.77)	2.0	1.92(1.11–3.33)	<0.001
HIV/ART status						
HIV negative	6,588	5.3	1	1.0	1	
HIV+ <5 years on ART	220	19.1	4.46(3.13–6.36)	5.5	6.56(3.48–12.35)	
HIV+ ≥5 years on ART	154	10.4	2.09(1.23–3.55)	2.0	2.01(0.63–6.49)	
Not on ART	218	19.3	4.43(3.11–6.32)	4.1	4.89(2.40–9.97)	
Unknown HIV status	1,745	6.0	1.13(0.90–1.42)	1.0	1.01(0.60–1.70)	<0.001

^1^ p values presented are from chi squared test for the univariate association of anemia level (grouped as no, mild and moderate-to-severe) with each explanatory variable in turn

**Table 5:** Distribution of anemia severity and multinomial logistic regression showing crude ORs of risk factors for mild and moderate-to-severe anemia and respective p values in women

		Mild anemia		Moderate-to-severe anemia		
Variable	No of women	Prevalence (%)	Crude OR (95% CI)	Prevalence (%)	Crude OR (95% CI)	*P* value^1^
Residence						
Rural (Karonga)	7,111	12.0		7.5	1	
Urban (Lilongwe)	7,867	13.5	1.17(1.07–1.29)	9.8	1.37(1.22–1.54)	<0.001
Age group (years)						
18–24	3,952	12.1	1	7.8	1	
25–34	4,983	12.7	1.06(0.94–-1.21)	8.3	1.07(0.91–1.25)	
35–44	2,844	14.5	1.31(1.13–1.51)	11.7	1.61(1.37–1.90)	
45–54	1,531	13.3	1.15(0.96–1.37)	10.1	1.34(1.09–1.64)	
55–64	912	11.1	0.87(0.70–1.10)	4.9	0.60(0.43–0.83)	<0.001
> 65	756	11.5	0.93(0.72–1.18)	6.0	0.74(0.53–1.02)	
Smoking status						
Never smoker	14,917	12.7	1	8.7	1	
Past	36	11.1	0.80(0.28–2.26)	2.8	0.29(0.04–2.14)	
Current	25	28.0	2.70(1.11–6.57	8.0	1.13(0.26–4.92)	0.17
Alcohol use						
No	14,248	12.8	1	8.7	1	
Light/irregular	668	12.0	0.93(0.73–1.18)	9.1	1.05(0.80–1.38)	
Moderate/heavy	62	9.7	0.72(0.31–1.68)	8.1	0.89(0.35–2.24)	0.88
Education level						
No formal/illiterate	1,699	11.8	1.02(0.87–1.21)	8.4	1.07(0.88–1.30)	
Standard	6,452	11.6	1	7.9	1	
Secondary	5,764	14.1	1.27(1.14–1.41)	9.0	1.19(1.05–1.35)	<0.001
Tertiary	1,063	14.5	1.37(1.14–1.66)	12.2	1.70(1.39–2.09)	
Occupation						
Unemployed	6,338	13.0	1	9.1	1	0.23
Farming/Fishing	4,765	12.0	0.89(0.80–1.00)	7.2	0.77(0.67–0.89)	
Self-employed	2,372	12.9	0.99(0.86–1.14)	8.9	0.97(0.82–1.15)	
Salaried worker	1,503	13.8	1.11(0.94–1.31)	8.4	1.30(1.08–1.56)	<0.001
Wealth quintile						
1^st^ (lowest)	2,742	12.0	1	7.4	1	
2^nd^	3,192	12.4	1.03(0.88–1.21)	7.2	0.97(0.79–1.18)	
3^rd^	2,874	12.2	1.03(0.88–1.21)	8.7	1.18(0.98–1.44)	
4^th^	3,531	12.9	1.11(0.96–1.30)	9.6	1.34(1.11–1.60)	
5^th^ (highest)	2,557	14.2	1.27(1.08–1.49)	10.6	1.53(1.27–1.86)	<0.001
HIV/ART status						
HIV negative	10,990	11.7	1	6.8	1	
HIV+ <5 years on ART	650	18.6	1.98(1.60–2.44)	16.0	2.92(2.33–3.67)	
HIV+ ≥5 years on ART	340	13.8	1.34(0.98–1.85)	14.7	2.46(1.80–3.36)	
HIV+ not on ART	495	20.8	2.49(1.98–3.15)	21.2	4.37(3.45–5.53)	
Unknown HIV status	2,493	14.1	1.31(1.16–1.49)	11.5	1.85(1.60–2.14)	
Pregnant						
No	14,211	12.2	1	8.5	1	
Yes	767	22.6	2.23(1.86–2.67)	11.9	1.69(1.34–2.13)	
Parity						
None	1,860	14.0	1.28(1.09–1.50)	12.2	1.70(1.42–2.03)	<0.001
1–2	4,369	11.9	1	7.8	1	
3–5	5,321	13.0	1.12(0.99–1.26)	8.8	1.16(1.00–1.34)	
6+	3,406	12.9	1.10(0.96–1.26)	7.6	0.98(0.82–1.16)	<0.001

^1^ p values presented are from chi squared test for the univariate association of anemia levels (grouped as no, mild and moderate-to-severe anemia) with each explanatory variable in turn

Adjusted ORs for each severity of anemia are presented in Tables 6 and 7.

**Table 6:** Multivariable analysis of anemia risk factors using multinomial regression among men

Variable	Mild anemia OR (95% CI)	P value^1^	Moderate-to-severe anemia OR (95% CI)	*P* value^1^
Age group (years)				
18–24	1		1	
25–34	1.44(1.01–2.03)	0.04	1.03(0.46–2.31)	0.95
35–44	1.97(1.38–2.82)	<0.001	1.72(0.78–3.77)	0.18
45–54	3.38(2.35–4.86)	<0.001	3.58(1.66–7.71)	0.001
55–64	4.78(3.29–6.95)	<0.001	3.40(1.46–7.91)	0.004
> 65	7.81(5.62–-10.86)	<0.001	7.95(4.04–15.62)	<0.001
Smoking status				
Never smoker	1		1	
Past	1.03(0.72–1.47)	0.87	1.09(0.51–2.32)	0.82
Current	1.24(0.94–1.64)	0.12	1.61(0.87–2.94)	0.12
Alcohol use				
No	1		1	
Light/irregular	0.92(0.75–1.14)	0.49	0.50(0.30–0.83)	0.008
Moderate/heavy	0.72(0.50–1.03)	0.07	0.31(0.11–0.89)	0.03
Residence				
Rural (Karonga)	1		1	
Urban (Lilongwe)	1.08(0.84–1.38)	0.57	0.92(0.52–1.62)	0.78
Education level				
No formal/illiterate	1.03(0.72–1.48)	0.88	1.81(0.94–3.46)	0.08
Standard	1		1	
Secondary	0.91(0.74–1.13)	0.39	1.06(0.66–1.70)	0.81
Tertiary	0.64(0.43–0.95)	0.03	1.93(0.91–4.11)	0.09
Occupation				
Unemployed	1		1	
Farming/Fishing	0.91(0.67–1.23)	0.53	0.98(0.53–1.81)	0.95
Self-employed	0.80(0.58–1.10)	0.17	0.97(0.51–1.83)	0.91
Salaried worker	0.82(0.61–1.10)	0.19	0.32(0.14–0.71)	0.005
Wealth quintile				
1^st^ (lowest)	1		1	
2^nd^	0.65(0.49–0.86)	0.002	1.00(0.56–1.79)	0.99
3^rd^	0.96(0.73–1.27)	0.77	0.95(0.51–1.77)	0.87
4^th^	0.82(0.62–1.10)	0.19	0.74(0.38–1.44)	0.37
5^th^ (highest)	0.71(0.50–1.00)	0.05	0.66(0.30–1.46)	0.31
Underweight				
No	1		1	
Yes	1.87(1.44–2.43)	<0.001	1.46(0.82–2.60)	<0.19
HIV/ART status				
HIV Negative	1		1	
HIV+ and <5 years on ART	3.59(2.48–5.22)	<0.001	5.16(2.63–10.12)	<0.001
HIV+ and >5 years on ART	1.36(0.78–2.35)	0.28	1.23(0.37–4.10)	0.73
HIV+ Not on ART	4.51(3.09–6.58)	<0.001	5.35(2.53–11.28)	<0.001
Unknown HIV status	1.24(0.97–1.57)	0.09	1.17(0.68.2.02)	<0.57

^1^ Wald test p values

**Table 7:** Multivariable analysis of anemia risk factors using multinomial regression among women

Variable	Mild anemia OR (95%CI)	P value^1^	Moderate-to-severe anemia OR (95%CI)	*P* value^1^
Age group (years)				
18–24	1		1	
25–34	1.09(0.93–1.29)	0.28	1.32(1.09–1.60)	0.005
35–44	1.38(1.12–1.68)	0.002	2.10(1.66–2.66)	<0.001
45–54	1.27(1.00–1.62)	0.05	1.86(1.40–2.45)	<0.001
55–64	0.95(0.71–1.27)	0.74	0.88(0.60–1.28)	0.49
> 65	1.08(0.80–1.48)	0.60	1.14(0.77–1.68)	0.53
Smoking status				
Never smoker	1		1	
Past	0.73(0.26–2.10)	0.57	0.24(0.03–1.82)	0.17
Current	2.65(1.07–6.57)	0.04	1.05(0.23–4.70)	0.95
Residence				
Rural (Karonga)	1		1	
Urban (Lilongwe)	1.08(0.92–1.26)	0.34	1.07(0.89–1.29)	0.46
Education level				
No formal/illiterate	1.02(0.85–1.21)	0.84	1.15(0.93–1.41)	0.19
Standard	1		1	
Secondary	1.31(1.15–1.48)	<0.001	1.02(0.88–1.19)	0.78
Tertiary	1.30(1.03–1.63)	0.03	1.12(0.87–1.44)	0.39
Wealth quintile				
1^st^ (lowest)	1		1	
2^nd^	1.03(0.88–1.21)	0.69	0.95(0.78–1.16)	0.63
3^rd^	0.97(0.82–1.15)	0.76	1.07(0.87–1.31)	0.54
4^th^	1.01(0.86–1.19)	0.91	1.13(0.93–1.38)	0.22
5^th^(highest)	1.10(0.92–1.32)	0.29	1.18(0.95–1.47)	0.13
Occupation				
Unemployed	1		1	
Farming/Fishing	1.05(0.88–1.25)	0.59	0.99(0.80–1.22)	0.90
Self-employed	0.97(0.83–1.13)	0.72	0.97(0.81–1.16)	0.72
Salaried worker	0.99(0.82–1.18)	0.88	1.04(0.85–1.28)	0.69
HIV/ART status				
HIV Negative	1		1	
HIV+ and <5 years on ART	2.01(1.62–2.48)	<0.001	2.82(2.24–3.65)	<0.001
HIV+ and >5 years on ART	1.30(0.94–1.80)	0.12	2.23(1.61–3.09)	<0.001
HIV+ not on ART	2.53(1.99–3.21)	<0.001	4.19(3.29–5.35)	<0.001
Unknown HIV status	1.22(1.06–1.39)	0.004	1.70(1.46–1.98)	<0.001
Currently pregnant				
No	1		1	
Yes	2.65(2.19–3.20)	<0.001	2.21(1.72–2.82)	<0.001
Parity				
None	1.43(1.19–1.72)	<0.001	2.11(1.72–2.60)	<0.001
1–2	1		1	
3–5	1.09(0.94–1.27)	0.23	0.94(0.79–1.11)	0.46
6+	1.25(1.02–1.53)	0.03	0.88(0.69–1.11)	0.28

^1^ Wald test p values are presented here

#### Men

Compared to HIV-negative men, HIV-positive men not on anti-retroviral treatment (ART) and those on ART for less than 5 years had highest odds of mild and moderate-to-severe anemia respectively. Underweight men had higher odds of mild anemia (OR: 1.87; 95% CI:1.44, 2.43). Reported alcohol use was associated with lower odds of having moderate-to-severe anemia for both light (OR: 0.50; 95% CI: 0.30, 0.83) and moderate-to-heavy (OR: 0.31; 95% CI: 0.11, 0.89) use.

#### Women

Positive HIV status was a strong risk factor across all levels of anemia. Compared to HIV-negative women, HIV-positive women, irrespective of their treatment status had higher odds of moderate-to-severe anemia. Pregnant women had more than twice the odds of mild (OR: 2.65; 95% CI: 2.19, 3.20) and moderate-to-severe (OR: 2.21; 95% CI: 1.72, 2.82) anemia. Compared to women with one to two previous pregnancies, nulliparous women had higher odds of both mild and moderate-to-severe anemia, while women with six or more previous pregnancies had higher odds of mild anemia.

### Population attributable fractions

We calculated proportions of mild and moderate anemia attributable to HIV infection (irrespective of ART treatment) as well as pregnancy (for women). Proportions of mild and moderate-to-severe anemia attributable to HIV were 15.2 and 19.6% among men; and 8.8 and 17.9% among women respectively. Proportions of mild and moderate-to-severe anemia attributable to current pregnancy in women were 6.1 and 3.9%, respectively.

## Discussion

Anemia is of moderate public health importance among women, and of mild public health importance in men, in these Malawian rural and urban populations, based on the WHO prevalence-based classification [28]. Multivariable analyses indicate that for men, increasing age and HIV infection were independent risk factors for both mild and moderate-to-severe anemia. Being underweight was also associated with mild anemia. Among women, being between 35 and 54 years of age, pregnancy, nulliparity, and HIV infection were risk factors for any anemia severity. Additionally, women with secondary or tertiary education and those with six or more previous pregnancies had higher odds of having mild anemia.

The prevalence of anemia in non-pregnant and pregnant women reported here is lower than that reported by the Malawi DHS. The DHS may exclude relatively healthier participants not found at home or the population may differ [24]. The differences in anemia prevalence between men and women was minimal after 55 years of age, and after 65 years prevalence was higher in men. This possibly indicates greater biological and social vulnerability during the reproductive years in women and among elderly men [31–33]. It is also plausible that interventions targeted at women during the reproductive years may protect them from anemia at later ages. This is also supported by the higher risk of anemia found among women who have never been pregnant although this may be related to fitness for reproduction.

Higher anemia prevalence in rural regions has been attributed to disparities in health service provision and access, disease risk, fertility preferences, and genetic conditions such as sickle cell anemia [16, 34–36]. Although urban/rural residence was not found to be an independent risk factor for anemia of any severity in men or women, crude rates of anemia were higher in urban than rural women but higher in rural than urban men. There were also urban/rural differences in types of anemia. Microcytic anemia was more prevalent among urban residents. Iron deficiency may be less prevalent in rural areas as rural residents have better access to diverse and more nutritious food sources; foods rich in iron such as eggs, leafy green vegetables and in our lake-shore population, fish; and fruits containing Vitamin C, enhancing iron absorption. Rural women may be more likely to access the mineral-rich clay pellets traditionally consumed during pregnancy [37].

Anemia is related inversely to economic development at country/regional level, to wealth at household level and to income and education at individual level [16, 24, 38]. Though studies have shown associations between low socioeconomic status and all anemia levels, our findings indicate that women with higher educational levels had higher odds of mild anemia [32, 34, 39]. We observed gender-specific variations in prevalence of anemia among different occupation groups. Amongst men, farmers/fishermen had the highest prevalence and amongst women, salaried workers. This indicates that the difference in drivers of anemia in men and relate to factors that affect production as well as breakdown or loss of red blood cells. Prevalence of microcytic anemia increased with increasing levels of education and was lowest among farmers. It is plausible that diets of more educated women from wealthier homes, as with urban residents, may have moved away from traditional diets and supplements, thereby increasing their risk of nutrition-related anemia. Conversely, farming/fishing can increase the risk of other anemia-inducing infectious diseases such as schistosomiasis and hookworm, and such exposures may be more common in men.

Repeated pregnancies and short birth intervals deplete iron stores, resulting in anemia, explaining the association of higher parity with mild anemia [7, 40, 41]. In contrast, higher parity was not associated with moderate-to-severe anemia. Interventions for anemia control are usually targeted at pregnant women, so multiparous women repeatedly access anemia-control interventions such as iron/folate supplement, insecticide-treated nets (ITN), malaria chemoprophylaxis, deworming, HIV testing, and subsequent treatment. This may buffer the effect of parity on anemia and maintain hemoglobin levels at mild anemia stage. High risks of mild and moderate-to-severe anemia among nulliparous women could be related to their physical fitness to reproduce and to associated co-morbidities. Although HIV infection and treatment status have been adjusted for, residual confounding is still possible from severity/stage of HIV which is unaccounted for in this analysis.

Low BMI (<18.5 kg/m^2^) was associated with mild anemia in men. Many studies have shown inverse relationships between BMI and anemia [31, 32, 34, 42]. A multi-country, multi-level analysis of hemoglobin levels of African women showed stronger associations of anemia with socioeconomic and contextual factors than with BMI [11]. Micronutrient deficiencies have been shown to have different roles in etiology of varying severities of anemia [17]. Iron and folate deficiency have been associated with mild anemia, while vitamins A and B12 deficiency have been shown to be important in the etiology of severe anemia [6, 17]. Although high BMI may not necessarily imply better micronutrient intake, underweight persons are more likely to have other associated comorbid illnesses and hence poorer nutrition.

Our results show that reported alcohol use strongly lowered the odds of moderate-to-severe anemia among men, even after adjustment for age, education, residence, occupation, BMI, smoking, and HIV status. There is some evidence that traditionally brewed beers in SSA have a high enough iron content to be protective against iron deficiency [43, 44]. However, due to the social nature of alcohol use, the role of reverse causality cannot be ruled out.

A strong association was observed with all severities of anemia and HIV. Anemia is a frequent complication in HIV infection and it has been shown to increase with disease progression [45, 46]. Use of ART has been shown to reduce anemia prevalence among HIV-infected, [47, 48] but our findings indicate that in this setting where anemia may not be actively managed in ART clinic attenders, this benefit may only be seen after several years of treatment. Conversely, use of certain ART medications (Zidovudine) may be associated with increased risk of anemia, through myelosuppression [47, 49]. Despite the moderate HIV prevalence in this study, almost 20% of severe anemia in the population was attributable to HIV.

The burden and distribution of anemia in this population has several implications. As anemia has been shown to negatively affect physical capacity and reduce individual productive capability, decrease in household income and food security is a likely consequence, especially in populations such as ours, that rely primarily on subsistence farming/fishing and other often-seasonal sources of income [16, 50]. Anemia can contribute to poor pregnancy and birth outcomes in women of reproductive age and these can worsen already poor maternal and perinatal indices [16] and may counteract the improved outcomes otherwise seen in educated urban women. Anemia among HIV-infected persons is associated with poorer quality of life, disease progression, and increased mortality; and therefore, can worsen prognosis, [45, 51] but may not be routinely screened for in all HIV services.

Our research findings should be considered in light of several limitations. The cross-sectional nature of the survey makes it difficult to explore temporal relationships. Though misclassification of exposure variables is likely to be minimal, exposure measured at time of survey may not reflect past exposure that may be important for a chronic condition such as anemia. Previous research has shown that place of long-term childhood residence may influence anemia risk more than place of current residence and the complete effect of SES on health may not be captured by measuring SES at a single point in time [32, 52, 53]. Finally, the large number of missing HIV and anemia observations may have introduced bias in to the sampling. The response rates in urban men were poor, which might introduce selection bias as employed men were challenging to recruit [27]. To minimize missing HIV data, we assessed HIV status from different sources – testing from current or prior study and self-report. Though it is unlikely that subjects will falsely report being HIV-positive, it is possible that participants that did not consent to HIV testing are more likely be HIV-positive. Thus, we may have underestimated the prevalence of anemia and the effect of HIV. Additionally, we may have overestimated the prevalence of anemia if healthier participants with lower risk of anemia had missing values because they were at work, and thus were unavailable for HIV testing. It is also likely that some of the women self-reporting as not pregnant may in fact have been in early pregnancy and this may have led to overestimation of anemia prevalence among non-pregnant women and underestimated the effect of pregnancy. The main limitation of our study is the absence of data on other anemia-inducing comorbidities. Underlying presence of infectious (malaria, schistosomiasis, and hookworm) and non-infectious (chronic kidney disease, menorrhagia, gastritis, peptic ulcer disease) diseases which are important in anemia etiology in SSA populations may have resulted in some residual confounding. For instance, because pregnant women are at particular risk of malaria, some of the observed effect of pregnancy may be confounded by malaria. The burden of some anemia-inducing chronic diseases such as chronic kidney disease and peptic ulcer disease may be higher in urban populations, and may contribute to the observed higher risk of anemia among urban women.

## Conclusion

This study provides several insights into the population burden of anemia among less well-studied groups and indicates that there may be risks associated with higher socioeconomic status and urban living. Biological (age, parity, pregnancy, and HIV infection) and social (urban residence, education, occupation, and wealth) factors are associated with anemia and this understanding can inform intervention strategies both in identification and management of high-risk groups as well as in the nature of the intervention. To effectively control anemia, a comprehensive and multi-sectoral approach will have far-reaching consequences. Supporting women farmers/fisherwomen will not only improve their nutritional status and reduce their nutrition-related anemia risk, but will improve the overall household income and promote empowerment of women and gender equality. Among urban women and women with higher socioeconomic status, food-based interventions such as dietary diversification and fortification of staple foods would reduce nutrition-related anemia as well as other micro-nutrient deficiencies. Consistent findings are the implications of HIV infection for higher risk of any severity of anemia, which may be contributing to poorer HIV outcomes. Wider access to ART and improved detection is important to control anemia among HIV-infected persons.

## Abbreviations

ART: Anti-retroviral treatment
BMI: Body mass index
DHS: Demographic and Health Survey
DSS: Demographic surveillance site
HCT: HIV counselling and testing
HIV: Human immunodeficiency virus
INACG: International Nutritional Anemia Consultative Group
ITN: Insecticide treated nets
LRT: Likelihood ratio test
MCHC: Mean cell hemoglobin concentration
MCV: Mean cell volume
MEIRU: Malawi epidemiology and intervention research unit
MNL: Multinomial logistic
OR: Odds ratio
SD: Standard deviation
SES: Socioeconomic status
SSA: Sub-Saharan Africa
WHO: World Health Organisation
